# Targeting HSF1 disrupts HSP90 chaperone function in chronic lymphocytic leukemia

**DOI:** 10.18632/oncotarget.5167

**Published:** 2015-09-10

**Authors:** Siddhartha Ganguly, Trisha Home, Abdulraheem Yacoub, Suman Kambhampati, Huidong Shi, Prasad Dandawate, Subhash Padhye, Ashok K. Saluja, Joseph McGuirk, Rekha Rao

**Affiliations:** ^1^ The University of Kansas Cancer Center, Kansas City, KS, USA; ^2^ Georgia Regents University, Augusta, GA, USA; ^3^ Interdisciplinary Science and Technology Research Academy, University of Pune, Maharashtra, India; ^4^ University of Minnesota, Minneapolis, MN, USA

**Keywords:** HSF1, HSP90, CLL, minnelide

## Abstract

CLL is a disease characterized by chromosomal deletions, acquired copy number changes and aneuploidy. Recent studies have shown that overexpression of Heat Shock Factor (HSF) 1 in aneuploid tumor cells can overcome deficiencies in heat shock protein (HSP) 90-mediated protein folding and restore protein homeostasis. Interestingly, several independent studies have demonstrated that HSF1 expression and activity also affects the chaperoning of HSP90 kinase clients, although the mechanism underlying this observation is unclear. Here, we determined how HSF1 regulates HSP90 function using CLL as a model system. We report that HSF1 is overexpressed in CLL and treatment with triptolide (a small molecule inhibitor of HSF1) induces apoptosis in cultured and primary CLL B-cells. We demonstrate that knockdown of HSF1 or its inhibition with triptolide results in the reduced association of HSP90 with its kinase co-chaperone cell division cycle 37 (CDC37), leading to the partial depletion of HSP90 client kinases, Bruton's Tyrosine Kinase (BTK), c-RAF and cyclin-dependent kinase 4 (CDK4). Treatment with triptolide or HSF1 knockdown disrupts the cytosolic complex between HSF1, p97, HSP90 and the HSP90 deacetylase- Histone deacetylase 6 (HDAC6). Consequently, HSF1 inhibition results in HSP90 acetylation and abrogation of its chaperone function. Finally, tail vein injection of Mec-1 cells into Rag2−/−IL2Rγc−/− mice followed by treatment with minnelide (a pro-drug of triptolide), reduced leukemia, increased survival and attenuated HSP90-dependent survival signaling *in vivo*. In conclusion, our study provides a strong rationale to target HSF1 and test the activity of minnelide against human CLL.

## INTRODUCTION

Chronic Lymphocytic Leukemia (CLL) is the most common adult leukemia in the western hemisphere [1, 2]. While, novel therapies including treatment with ibrutinib, a Bruton's Tyrosine kinase (BTK) inhibitor, result in durable remissions in CLL, a proportion of patients still develop resistance to therapy [3]. CLL is characterized by the acquisition of one or more acquired or inherited chromosomal deletions or gains and single nucleotide polymorphisms (SNPs) [4]. Given the fact that aneuploidy and the resulting non-stoichiometric amounts of cellular proteins impair HSP90 function, we hypothesized that protein homeostatic mechanisms in aneuploid CLL are likely to be deregulated [5, 6].

A recent study has reported that overexpression of Heat Shock Factor 1 (HSF1) restores the ability of aneuploid cells to maintain protein homeostasis [6, 7]. HSF1, a stress-inducible transcription factor, exists in a repressive complex comprising HSP90, HDAC6 and p97 (a segregase with ATPase activity) [8]. Heat shock, accumulation of misfolded proteins, or malignant transformation, results in the dissociation of HSF1 from the repressive complex. Activated HSF1 up-regulates the transcription of HSPs, which ameliorate misfolded protein-induced proteotoxic stress. HSF1-induced heat shock proteins (HSPs) promote survival of cancer cells following exposure to chemotherapeutic agents and have been implicated in conferring resistance to chemotherapy as well as promotion of tumor growth and metastasis [9–11].

HSP90 and its associated co-chaperones (HSP70, CDC37, p23, AHSA1, etc) maintain protein homeostasis in all cells by promoting the folding and maturation of meta-stable HSP90 client proteins [12]. The HSP90 co-chaperone, HSP70 promotes the proteasomal degradation of misfolded HSP90 client proteins, thereby facilitating their disposal. Consequently, agents that promote proteotoxic stress such as HSP90 inhibitors preferentially target tumor cells, which are typically “addicted” to HSP90 chaperone function [13, 14]. Furthermore, several kinase oncoproteins (BCR-ABL, NPM-ALK) and survival kinases (c-RAF, CDK4, BTK) are chaperoned by the HSP90-CDC37 complex [15–18]. Consequently, tumor cells can be selectively targeted by inhibiting HSP90-CDC37 interaction or down-regulation of CDC37 [18–20]. CD19+ CLL B cells, in particular, are dependent on HSP90-dependent kinases such as BTK, zeta-chain associated protein kinase 70 kDa (ZAP70) and AKT, among several others, for their survival and proliferation [21, 22]

Triptolide, a small molecule inhibitor of HSF1, is a diterpenoid epoxide isolated from the Chinese herb *Tripterygium wilfordii*. Triptolide and its derivatives have demonstrated anti-cancer activity against several pre-clinical models of pancreatic cancer, ovarian cancer, breast cancer, acute myeloid leukemia, multiple myeloma and osteosarcoma [23–27]. Treatment with triptolide results in the transcriptional inhibition of HSP70 mRNA and the upregulation of miR-142-3p, a microRNA that downregulates HSP70 mRNA levels. Several alternative mechanisms of triptolide action have been described including activation TRAIL-induced death receptor pathway, induction endoplasmic reticulum (ER) stress and inhibition SP-1 transcriptional activity as well as NF-kappa B activity [28–31]. A water-soluble pro-drug of triptolide called minnelide has shown demonstrable pre-clinical activity against pancreatic cancer, ovarian cancer, osteosarcoma and hepatocellular carcinoma [25, 26]. Currently minnelide is being evaluated in a phase 1 clinical trial (NCT01927965) against advanced human gastrointestinal tumors.

Considering the fact that HSF1 overexpression promotes HSP90 chaperone function in aneuploid cancer cells and the fact that CLL is characterized by one or more chromosomal abnormalities, we determined whether HSF1 is overexpressed in CLL [4, 32, 33]. We report the overexpression of HSF1 in CLL and demonstrate that inhibition of HSF1 function with triptolide results in a dose-dependent increase in apoptosis of CLL cells. It has been reported that HSF1 deletion impairs the chaperoning of numerous HSP90 kinase client proteins including c-RAF, AKT and MIF (macrophage migration inhibitory factor) [34, 35]. However, it has not been clear how HSF1 mechanistically inhibits HSP90 chaperone function. Here, we demonstrate that triptolide inhibits HSP90-CDC37 binding and induces acetylation of HSP90. Consequently, treatment with triptolide (*in vitro*) or minnelide (*in vivo*) disrupts HSP90 chaperone function and BTK-PLC-γ2 signaling. Collectively, our findings suggest that HSF1 promotes tumorigenesis at least in part by affecting HSP90-dependent B-cell survival signaling in CLL.

## RESULTS

### HSF1 is overexpressed in CLL

**Figure 1:**
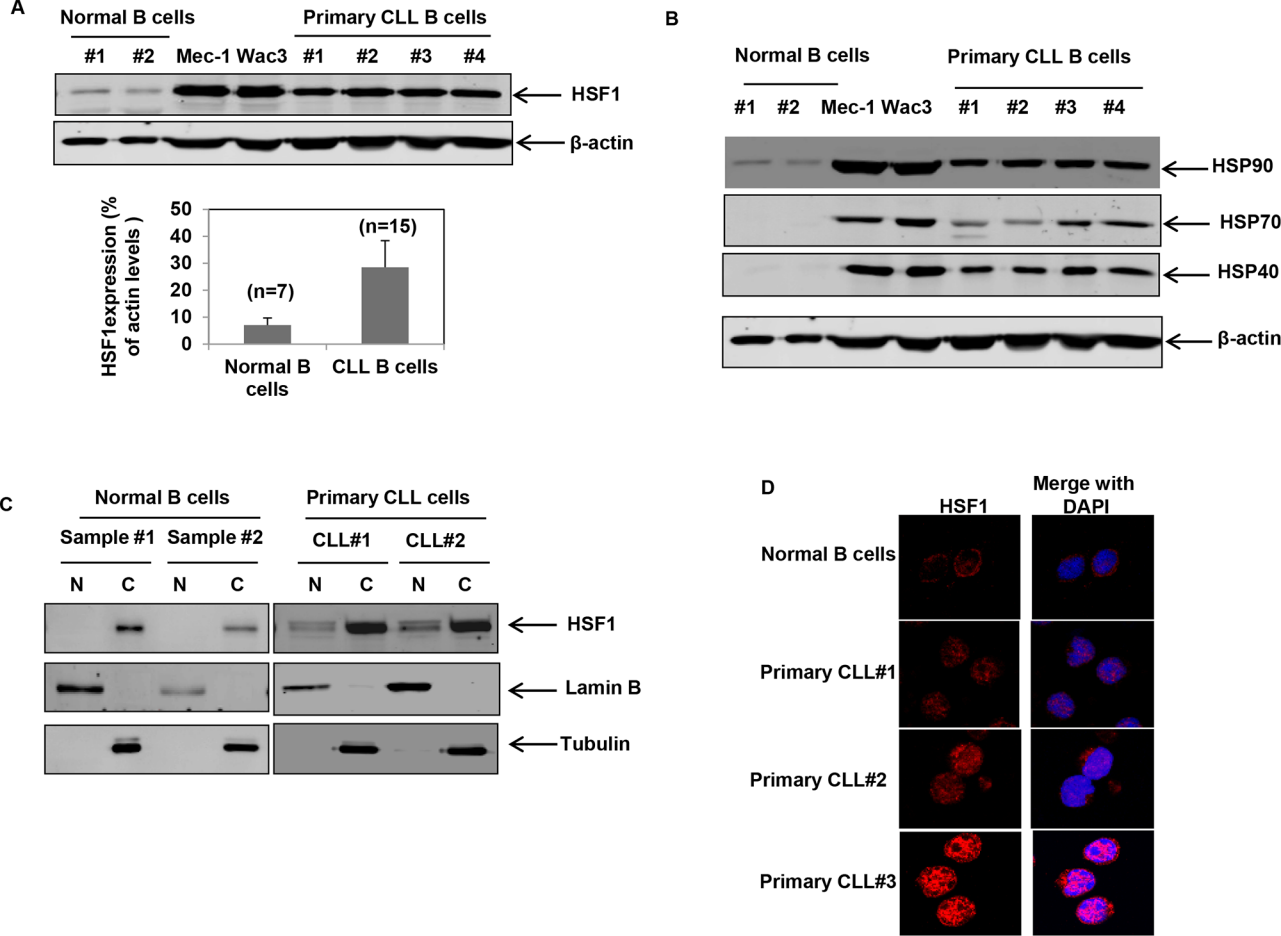
HSF1 is over-expressed in CD19+ primary CLL cells and cultured CLL cells **A.** Immunoblot analyses of HSF1 and β-actin in the cell lysates of CD19+ normal B cells, cultured CLL cells (Mec-1 and WaC3-CD5+) and CD19+ primary CLL cells. Quantitation of HSF1 levels in normal (*n* = 7) versus CLL B cells (*n* = 16) is provided in the bar chart below. Bars indicate standard error of the mean. Relative HSF1 levels are expressed as a percent of total cellular β-actin levels. **B.** Immunoblot analyses of heat shock proteins and β-actin in cultured CLL cells and CD19+ normal as well as primary CLL cells. **C.** Immunoblot analyses of HSF1 in the nuclear (N) and cytosolic (C) fractions of CD19+ normal B cells and primary CLL cells; Lamin B was used as the nuclear loading control and Tubulin was used as the cytosolic loading control. **D.** Representative immunofluorescent staining of HSF1 (in red) in normal B cells and primary CD19+ CLL cells.

Consistent with the upregulation of HSPs in cancer cells and CLL, both cultured and primary CLL B cells showed increased expression of HSF1 and the heat shock proteins HSP90, HSP70 and HSP40 compared to normal B cells (Figures 1A & 1B) [36]. The relative expression of HSF1 was significantly higher in CD19+ CLL B cells (*n* = 15) compared to normal B cells (*n* = 7) *p* = 0.0003 (Figure 1A, lower panel and supplementary Figure 1). The increased expression of HSF1 also correlated with an increase in the levels of HSPs in cultured and primary CLL B cells (Figure 1B). The expression of HSP27 varied widely with no clear evidence of overexpression in all CLL samples tested (data not shown). Evaluation of the localization of HSF1 by fractionation of cellular proteins into nuclear and cytosolic fractions revealed that a fraction of HSF1 (8–10%) is nuclear in CLL B cells as compared to a pre-dominant cytosolic localization of HSF1 in normal B cells (Figure 1C). These findings were also confirmed by confocal immunofluorescent staining to determine the localization of HSF1 in normal and CLL samples. Interestingly, HSF1 was localized in the nucleus of CLL patients stratified as low risk (indolent disease or requiring no treatment, CLL#1) or high risk (relapsed/refractory disease or requiring treatment, CLL#2 and CLL#3) (Figure 1D). Collectively, these observations suggest that HSF1 is overexpressed in CLL B cells.

### Treatment with triptolide induces apoptosis in cultured and primary CLL cells

**Figure 2:**
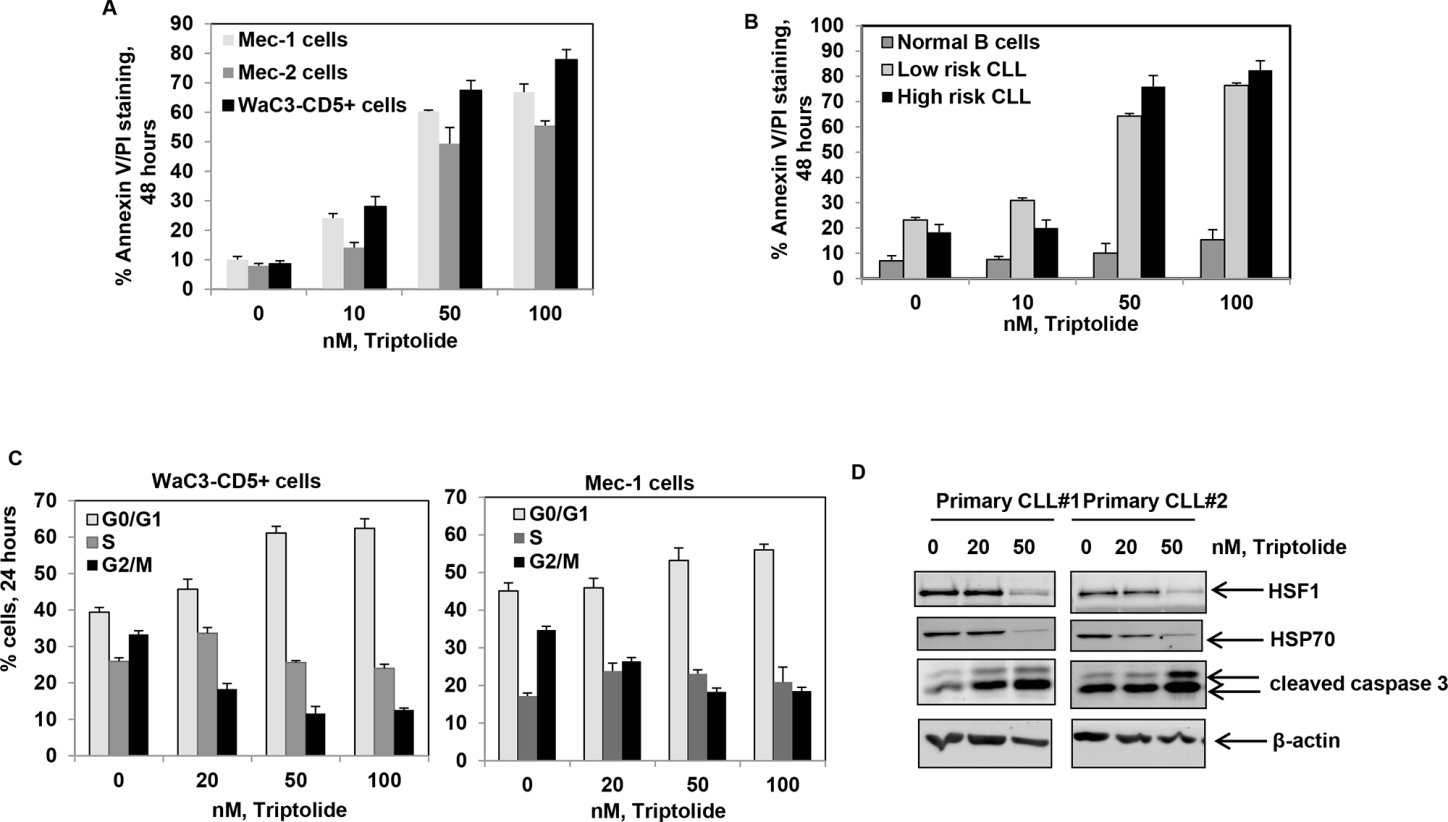
Treatment with triptolide selectively induces apoptosis of cultured and primary CD19+ CLL cells **A.** Percent apoptosis (Annexin V-FITC and PI staining) in Mec-1, Mec-2 and WAC3-CD5+ cells exposed to the indicated doses of triptolide for 48 hrs; bars indicate standard deviation. **B.** Percent apoptosis of CD19+ normal and primary CLL (both high and low risk) cells exposed to the indicated doses of triptolide for 48 hrs; bars indicate standard deviation. **C.** Percent of WaC3-CD5+ and Mec-1 cells in different phases of the cell cycle (G0/G1, S and G2/M) exposed to the indicated doses of triptolide for 24 hrs. **D.** Immunoblot analyses of HSF1, HSP70, cleaved caspase-3 and β-actin obtained from the cell lysates of CD19+ primary CLL cell samples treated with the indicated doses of triptolide for 24 hrs.

Having observed that HSF1 is overexpressed in CLL B cells, we next asked whether we could target HSF1 in CLL. Treatment of CD19+ B cells with triptolide, a small molecule inhibitor of HSF1 function, induced a dose-dependent increase in apoptosis in cultured and primary CLL cells. Triptolide was selectively toxic to both high risk (*n* = 5) and low risk CLL (*n* = 12) B cells (10 to 50 nM range) while largely sparing normal B-cells (*n* = 5) (Figure 2A and 2B). Consistent with the inhibition of heat-shock induced HSP transcription, treatment with triptolide attenuated heat-shock induced expression of HSPs (Supplementary Figure 2A). As noted previously in multiple myeloma and glioma, CLL cells accumulated in the G0-G1 phase of the cell cycle cell following triptolide treatment (Figure 2C) [37]. Finally, treatment with triptolide resulted in the depletion of HSP70 and the induction of Caspase-3 cleavage and PARP cleavage in cultured and primary CLL cells (Figure 2D and supplementary Figure 2B). These observations suggest that HSF1 inhibition has selective anti-CLL activity.

### Triptolide disrupts the association of HSP90 with CDC37 and results in the partial depletion of its kinase clients

**Figure 3:**
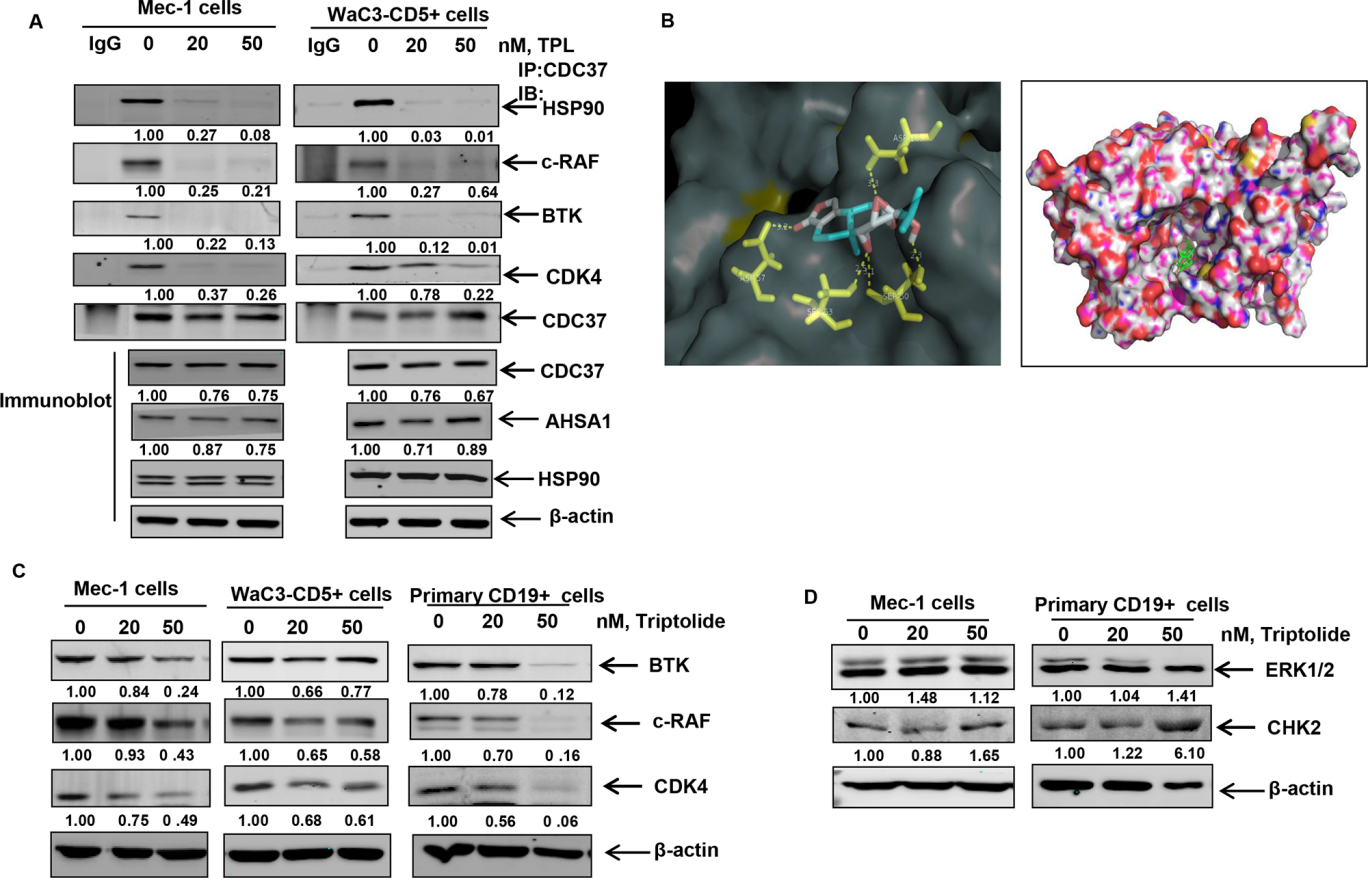
Triptolide treatment disrupts binding of HSP90 to CDC37 and HSP90 client proteins **A.** Immunoprecipitation of CDC37 from Mec-1 and WaC3-CD5+ cell lysates treated with triptolide for 16 hours, followed by immunoblot analyses of HSP90 and the client proteins BTK, c-RAF and CDK4. Bottom panel shows immunoblot analysis of CDC37, ASHA1 and total HSP90 from the respective total cell lysates. **B.** Molecular modeling of HSP90-CDC37 complex with triptolide as assessed by Autodock Vina software. **C.** Immunoblot analyses of selected client proteins BTK, c-RAF and CDK4 in cultured and primary CLL cells following exposure to the indicated doses of triptolide for 24 hrs. β-actin was loading control. The data presented are representative of three independent experiments. **D.** Immunoblot analysis of the non-client kinases CHK2 and ERK1/2 following treatment with triptolide for 24 hours.

Several studies have reported that genetic deletion of HSF1 results in reduced association of HSP90 with its kinase client proteins [34, 35]. However, the molecular basis of this observation has not been elucidated. Owing to the fact that most HSP90 kinase clients require the association of the co-chaperone CDC37 with HSP90 to promote their maturation, we determined whether triptolide affects the interaction of HSP90 with CDC37 [38]. Immunoprecipitation of CDC37 from triptolide-treated CLL cells revealed that triptolide treatment resulted in decreased association of HSP90 with CDC37. This was associated with the reduced interaction of CDC37 with HSP90 kinase clients BTK, c-RAF and CDK4 (Figure 3A). These findings were associated with no significant changes in the levels of CDC37, AHSA1 (an activator of HSP90 ATPase activity) or total HSP90 in the total cell lysates obtained from triptolide-treated Mec-1 and WaC3-CD5+ cells (Figure 3A bottom panel) [38].

In order to further corroborate our *in vitro* binding studies, we performed molecular docking of triptolide with the available crystal structure of HSP90-CDC37 [39]. Our studies revealed that consistent with the inhibition of interaction of CDC37 with HSP90, triptolide could be docked to both HSP90 with a binding energy of −6.7 Kcal/mol and to the HSP90-CDC37 complex with a binding energy of −9.4 Kcal/mol. We further determined that triptolide formed hydrogen bonds with Phe37 (at a distance of 3.4 Å), Asp127 (3.4 Å) of the N terminus domain of HSP90 alone and formed hydrogen bonds with residues Asp169 (3.3 Å) of CDC37 and residues Asp57 (32. Å), Ser53 (2.5 Å) and Ser50 (3.1 Å) of HSP90 (Figure 3B) in the HSP90-CDC37 complex. We determined that amino acid residues 117, 121, 123, 124, 125, 126 and 129 of HSP90 and 160, 161, 164, 165, 166, 167, 168, 193, 202, 204, 205 and 208 of CDC37 are among the key residues involved in HSP90-CDC37 interaction [39]. Our docking studies reveal that by interacting with critical residues on HSP90 and CDC37 (including Leu 205 as reported in earlier studies), triptolide can potentially bind to and disrupt the interaction of the HSP90-CDC37 complex [39]. Consequently we observed that treatment with triptolide resulted in the partial depletion of kinase clients of HSP90 such as BTK, c-RAF and CDK4 in cultured and primary CLL cells (Figure 3C), with no decrease in the levels of non-HSP90 client kinases, ERK1/2 and CHK2 (Figure 3D).

**Figure 4:**
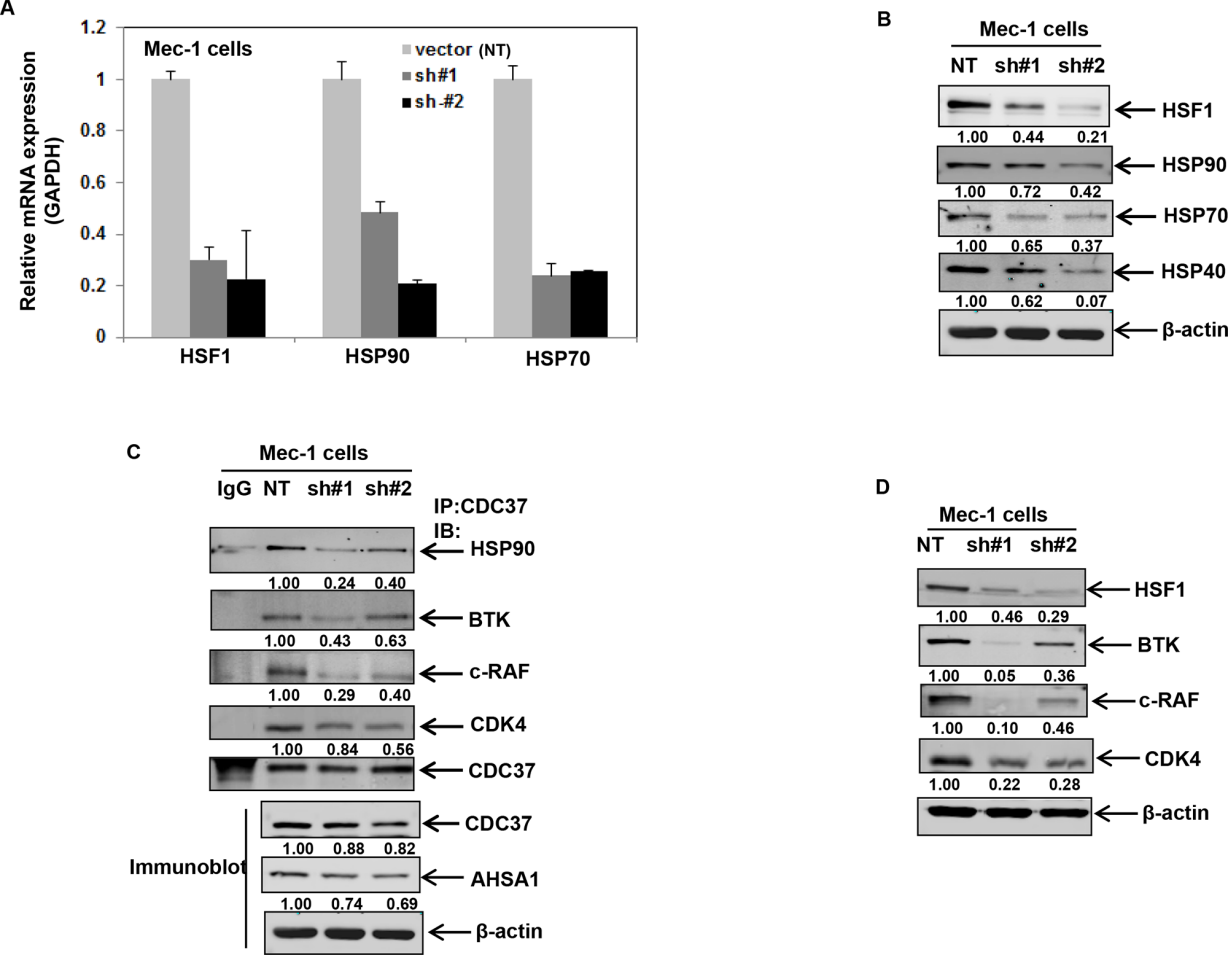
Stable shRNA-mediated knockdown of HSF1 disrupts binding of HSP90 to CDC37 and HSP90 client proteins **A.** mRNA expression of HSF1, HSP90 and HSP70 following knockdown of HSF1 with two independent HSF1 shRNA in Mec-1 cells. **B.** Stable knockdown of HSF1 followed by immunoblot analysis of HSF1 and HSPs. **C.** Immunoprecipitation of CDC37 in Mec-1 cell lysates with HSF1 knockdown followed by immunoblotting of HSP90 and the indicated HSP90 client proteins. Bottom panel shows the expression of CDC37 and AHSA1 from total cell lysates. **D.** Immunoblot analysis HSF1 and the indicated HSP90 client proteins in Mec-1 cell lysates obtained following stable knockdown of HSF1.

We then asked how knockdown of HSF1 affects the chaperone function of HSP90. shRNA-mediated stable knockdown of HSF1 in Mec-1 cells resulted in decreased expression of HSPs both at the mRNA and protein level (Figures 4A & 4B). Knockdown of HSF1 decreased the association of HSP90 with CDC37, with only a moderate decrease in the total levels of CDC37 and AHSA1 (Figure 4C bottom panel), ruling out the possibility that the observed effects on CDC37 binding were due to loss of CDC37/AHSA1 expression. Consequently, knockdown of HSF1 resulted in the partial depletion of HSP90 kinase clients- BTK, c-RAF and CDK4 (Figure 4D). These observations suggest that inhibition of HSF1 either by triptolide or HSF1 knockdown can affect HSP90-CDC37 association and the chaperoning of HSP90 kinase clients.

### Treatment with triptolide and knockdown of HSF1 disrupts the HSP90-HSF1-HDAC6-p97 complex and promotes HSP90 acetylation

**Figure 5:**
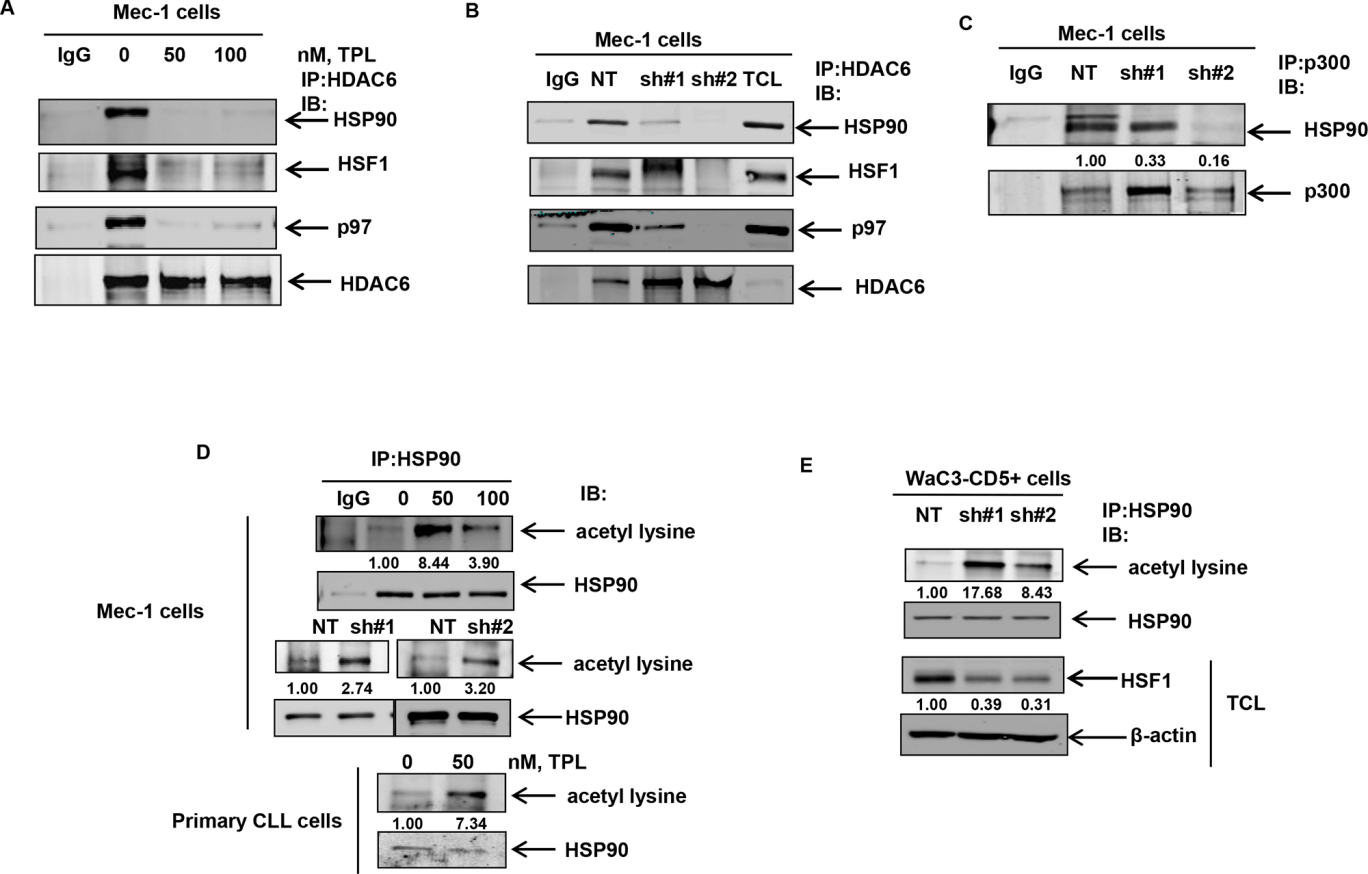
Treatment with triptolide or knockdown of HSF1 results in the disruption of the repressive complex and induces HSP90 acetylation **A.** and **B.** Immunoprecipitation of HDAC6 followed by immunoblot analyses of HSP90, HSF1, p97 and HDAC6 in Mec-1 cell lysates following treatment with the indicated doses of triptolide for 16 hrs or knockdown of HSF1. **C.** Immunoprecipitation of p300 from NT and shHSF1-infected Mec-1 cells followed by immunoblot analyses of HSP90. **D.** Immunoprecipitation of HSP90 from Mec-1 and primary CLL cells following treatment with triptolide for 16 hours or knockdown of HSF1 in Mec-1 cells followed by immunoblot analyses of acetylated lysine. The blots were stripped and re-probed for HSP90. **E.** Immunoprecipitation of HSP90 and immunoblot analyses of acetylated lysine from WaC3-CD5+ cell lysates following knockdown of HSF1 with two independent HSF1 shRNAs. Bottom panel shows immunoblot analyses of HSF1 and β-actin in the total cell lysates (TCL).

It has been reported that HSF1 is held in a “repressed” state in a complex comprising HSP90-HSF1-HDAC6-p97. We explored the possibility that reduced levels of HSF1 following HSF1 knockdown or treatment with triptolide (Figure 2D) could result in the disruption of the repressive complex [8]. We reasoned that non-stoichiometric levels of HSF1 might reduce the association of HSP90 with its deacetylase HDAC6 in the repressive complex, leading to HSP90 acetylation and inhibition of its chaperone function [40–44]. Indeed, acetylation-mimetic HSP90 (K/Q) mutants in the middle domain of HSP90 (K100, K292, K327, K478, K546, and K558) display reduced association of HSP90 with its co-chaperones and client proteins [40, 41]. Treatment with triptolide or stable knockdown of HSF1 disrupted the binding of HDAC6 with HSP90, p97 and HSF1 (Figure 5A & 5B). Reversible acetylation of HSP90 is regulated by the activity of the acetyl transferase (p300) and its deacetylase HDAC6 [40]. We therefore determined whether HSF1 knockdown affects the binding of HSP90 to p300. As shown in Figure 5C, knockdown of HSF1 results in the decreased binding of HSP90 to its acetyl transferase p300. Consequently, knockdown of HSF1 was associated with an increase in the acetylation of HSP90 in cultured and primary CLL cells (Figure 5D and 5E).

**Figure 6:**
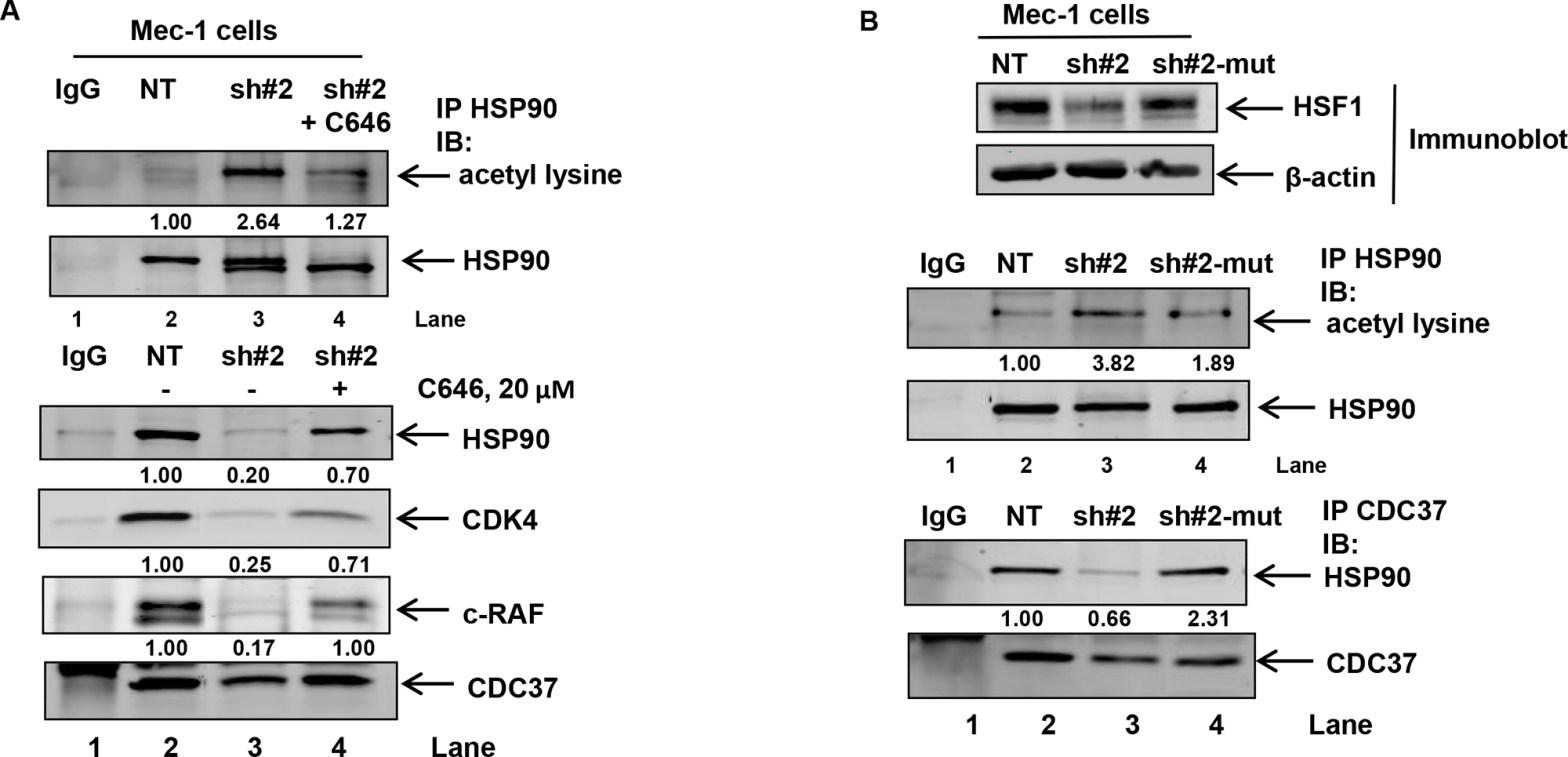
Treatment with the p300 inhibitor C646 or expression of shRNA insensitive HSF1 construct decreases HSF1-knockdown induced HSP90 acetylation and binding of HSP90 to CDC37 **A.** Immunoprecipitation of HSP90 from NT, shHSF1 or shHSF1 Mec-1 cells treated with 20 μM, C646 for 24 hours followed by immunoblotting for acetyl lysine and HSP90 (top panel). Immunoprecipitation of CDC37 followed by immunoblot analysis of HSP90 and the indicated HSP90 client kinases (bottom panel). **B.** Expression of shHSF1-insensitive HSF1 plasmid in shHSF1-transduced Mec1 cells followed by immunoprecipitation of HSP90 and CDC37 to assess HSP90 acetylation (top panel) as well as binding of CDC37 to HSP90 (bottom panel), respectively.

In order to assess whether p300 inhibition would reduce HSP90 acetylation under conditions where HDAC6 binding to HSP90 is also compromised, we treated HSF1 knockdown cells with C646, a small molecule inhibitor of p300 activity [45, 46]. Consistent with the notion that inhibition of p300 activity would decrease HSF1 knockdown-induced acetylation of HSP90, we observed that treatment with C646 resulted in a decrease in the extent of HSP90 acetylation (lane 4 versus lane 3) in shHSF1 infected Mec-1 cells (Figure 6A upper panel). This was associated with the restoration of CDC37 binding to HSP90 in HSF1 knockdown cells as well as its kinase clients, c-RAF and CDK4 (Figure 6A, lower panel). Furthermore, transient expression of an sh-RNA-insensitive HSF1 (sh#2-mut) plasmid in sh#2 Mec-1 cells restores HSF1 expression (Figure 6B upper panel) and decreases the extent of HSP90 acetylation (lane 4 versus lane 3) as well as restores HSP90 binding to CDC37 (Figure 6B lower panels). These observations collectively establish a causal link between A, HSF1 expression and HSP90 acetylation and B, HSF1 expression and the ability of HSP90 to bind to CDC37.

### Minnelide inhibits leukemogenesis in an *in vivo* model of CLL

**Figure 7:**
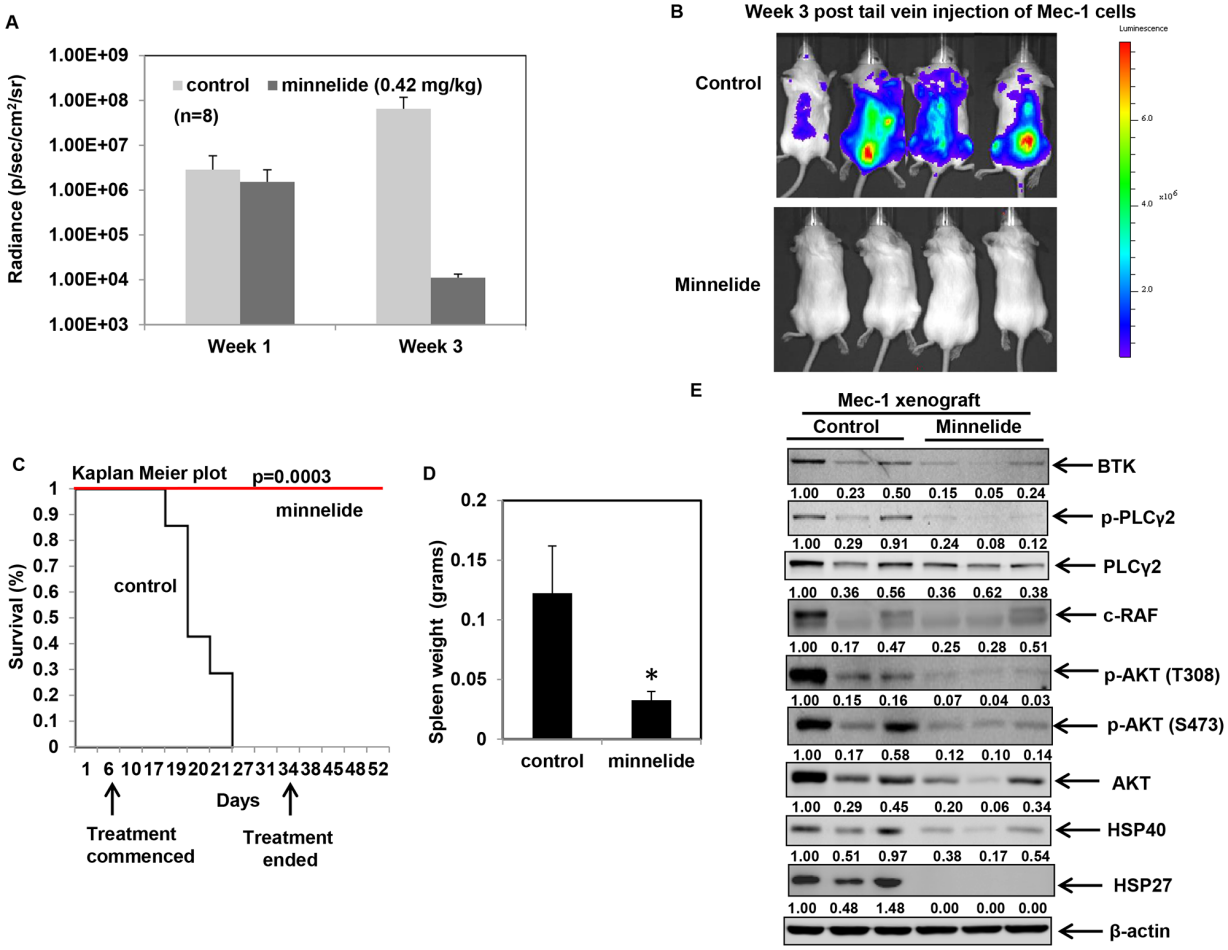
Minnelide treatment delays leukemogenesis and inhibits HSP90 function in an *in vivo* model of CLL **A.** Average radiance of the mice at the indicated times from an *in vivo* Mec-1luciferase expressing xenograft model of CLL in Rag2−/−IL2Rγ−/− mice (*n* = 8 per group) **B.** Bioluminescent images of control and minnelide-treated mice acquired at the same exposure conditions at week 3 following injection of Mec-1 luciferase cells. **C.** Kaplan Meier survival plots for control and minnelide-treated mice following indicated days of treatment. **D.** Spleen weight of deceased control mice and minnelide-treated mice on day 50 (*p = 0.04). **E.** Immunoblot analysis of BTK-PLCγ2-AKT signaling pathway and the indicated HSPs from control (from spleen collected at the time of death) and minnelide-treated mice (euthanized on day 50).

Having confirmed the *in vitro* activity of triptolide against CLL cells, we asked whether it could be used to impair CLL growth and maintenance *in vivo*. Owing to the reported toxicity of triptolide *in vivo*, we used minnelide, a pro-drug of triptolide to test its anti-CLL activity in an *in vivo* model of CLL [25]. Luciferase-expressing Mec-1 cells were injected into the tail vein of in Rag2−/−, IL2Rγ−/− mice to induce CLL, as previously described [47]. Treatment with minnelide commenced when the mice showed measurable average radiance of at least 1.00E+06 p/sec/cm2/sr and continued for 28 days. All mice were monitored regularly for disease progression by weekly bioluminescent imaging. As evident from Figures 7A and 7B, at week 1 post-injection of Mec1-luciferase cells, both control and minnelide treatment group of mice showed comparable induction of the disease. Treatment with minnelide resulted in a significant decrease in radiance compared to control mice by week 3. Furthermore, minnelide treatment conferred a significant survival advantage (*p* ≤ 0.0003) compared to control mice (Figure 7C) with the median survival for control mice being 19 days. Notably treatment with minnelide did not affect the body score of the animals (see supplementary Figure 3) throughout the course of the treatment. On day 50 (*p* < 0.0003 for Kaplan Meier analysis), three mice from the minnelide-treated group, were sacrificed to carry out correlative studies. The remaining minnelide-treated mice (*n* = 5) were alive even at day 60, after which the experiment was terminated. As noted in Figure 7D, minnelide-treated mice showed reduced spleen size compared to control mice (*n* = 3 per group). Consistent with the inhibition of HSF1/HSP90 function, treatment with minnelide resulted in a decrease in the levels of BTK in the treated-group. Considering the importance of B-cell receptor (BCR)-induced BTK-PLCγ2-AKT signaling cascade in CLL, we determined the effect of minnelide treatment on the phosphorylation of PLCγ2 and AKT [21]. Our data suggests that minnelide reduces the levels of p-PLCγ2 and p-AKT in the spleens of the treated mice (Figure 7E). Treatment with minnelide also resulted in a significant reduction in the levels of HSPs in the xenografts, consistent with the previously reported effects of minnelide on HSP70 [30]. Collectively, these observations suggest that inhibition of HSF1 *in vivo* decreases tumor burden and delays leukemogenesis. These pre-clinical findings create a strong rationale to test the efficacy of minnelide as a chemotherapeutic agent against CLL in humans.

## DISCUSSION

In this study we have identified HSF1 as a therapeutic target in indolent as well as aggressive CLL. We demonstrate for the first time that treatment with triptolide or depletion of cellular HSF1 levels disrupts the association of HSF1 with components of the cytosolic repressive complex comprising HSF1-HSP90-HDAC6-p97, resulting in the acetylation of HSP90. Consistent with the destabilization of HSP90 clients following HSP90 acetylation, we demonstrate that HSF1 inhibition (with triptolide) or depletion (by shRNA) abrogates the binding of HSP90 to its kinase client proteins c-RAF, BTK and CDK4 resulting in their partial depletion.

It is not clear not why shHSF1-#1 (50% HSF1 knockdown) causes greater depletion of the levels of HSP90 client proteins compared to shHSF1-#2 (70% knockdown) in Mec-1 cells. A possible explanation could be because HSP90 is more accessible to p300 binding in shHSF1-sh#1 compared to shSHF1-#2 Mec-1 cells (Figure 5C). Secondly, it is not clear if and how HSF1 inhibition/depletion affects the phosphorylation of CDC37 or HSP90, which would in turn affect the binding of CDC37 to its clients or HSP90, respectively [48, 49]. However, as has been reported following treatment with celastrol, a disruptor of HSP90-CDC37 complex, it is likely that triptolide might also affect HSP90 chaperone activity by covalently modifying CDC37 [50]. Unlike celastrol and geldanamycin analogues, triptolide does not induce a heat shock response, thus distinguishing its mechanism of action from that of celastrol or other geldanamycin analogues which result in the transcriptional up-regulation of HSPs [51]. The fact that triptolide directly binds to HSP90 at low nanomolar concentrations, suggests that HSP90 is a target of triptolide action.

We report that treatment with triptolide causes apoptosis in cultured and primary CLL cells. While HSF1 knockdown does not phenocopy treatment with triptolide in terms of inducing apoptosis, it does cause cell cycle arrest at the G0-G1 phase (for shHSF1-#2) or G1-S phase (for sh#1) (supplementary Figure 4) and inhibits B-cell survival signaling *in vivo*. These factors could adversely affect CLL cell survival as well as leukemogenesis *in vivo*. We demonstrate that treatment with minnelide, a pro-drug of triptolide, significantly improved survival in a mouse model of CLL utilizing Mec-1 cells. Minnelide is converted to triptolide *in vivo* by the action of alkaline phosphatases. As such, minnelide cannot be used *in vitro* without conversion into its parent compound as described previously [25]. Our findings point to the fact that minnelide is an effective anti-CLL agent when used as a monotherapy, with no observable toxicity, in contrast to reported toxic effects of triptolide. Considering the fact that many CLL patients live for prolonged periods without treatment, the ability of minnelide to delay disease progression in CLL (or possibly impact the natural history of the disease) could be translated clinically into testing its efficacy as a chemotherapeutic and/or a chemopreventive agent.

While most of the demonstrated effects of triptolide action have been the inhibition of HSP70 function or transcriptional inhibition of several tumor promoting genes, our studies highlight yet another aspect of its mechanism of action viz., HSP90 inhibition as evidenced by reduced *in vivo* BTK-PLCγ2-AKT signaling [11, 30, 52]. Based on our observation that triptolide is effective against relapsed/refractory CLL B cells, it is tempting to speculate that HSF1 inhibitors could also be used effectively against the entire spectrum of CLL patients, as it affects the BCR signaling cascade. Collectively our results provide a strong rationale to test the clinical efficacy of minnelide against human CLL.

## MATERIALS AND METHODS

### Ethics statement

The current study has been conducted in accordance with the Declaration of Helsinki as well as the national and international guidelines. This study has been approved by the authors’ institutional review board.

### Reagents, cell culture, isolation of primary CD19+ CLL cells and

Triptolide and C646 were purchased from Selleck chemicals. HSF1 expression plasmid (HSF1-GFPN3) was obtained from Addgene. Mec-1 cells were purchased from DSMZ. Mec-2 and WAC3-CD5+ cells were obtained from Dr. H. Shi (GRU Cancer Center, GA). All cells were cultured as described previously and authenticated by surface staining and flow cytometry for CD19, CD20 and CD79a [53]. Stable knockdown of HSF1 was carried out in Mec-1 and WaC3-CD5+ cells using lentiviral non-targeted or two independent HSF1 shRNA constructs (Sigma Aldrich, MO). De-identified and de-linked primary CLL samples were obtained from the Biorepository Core Facility of Kansas University Medical Center, after informed consent using an institutional review board-approved protocol (HSC-5929). CD19+ B cells from newly diagnosed, relapsed or treatment refractory CLL patients were isolated from the samples utilizing a magnetic CD19-positive selection kit (Stem Cell Technologies, Vancouver, BC), as described previously [54]. The purity of the isolated CD19+ B-cell fraction was assessed using CD19-PE conjugated antibodies (BD Biosciences, San Jose, CA) and flow cytometry. Positively selected cells were re-suspended in 20% FBS containing RPMI prior to performing the studies described.

### shRNA-mediated knockdown of HSF1 and transfection of shRNA-insensitive HSF1 mutant plasmid in Mec-1 cells

Knockdown of HSF1 was carried out in Mec-1 and WaC3-CD5+ cells using lentiviral non-targeted or two independent HSF1 shRNA constructs (Sigma Aldrich, MO). Stable clones were selected by growing shRNA-infected cells in puromycin. HSF1-GFPN3 construct was used as a template to obtain an shRNA-insensitive HSF1 (mutant) plasmid. For this purpose, the shHSF1-#2 sequence (GCCCAAGTACTTCAAGCACAA) was mutagenized utilizing the site-directed mutagenesis kit (QuikChange II site-directed mutagenesis kit, Agilent technologies Inc., CA) to the following sequence GCCTAAATACTTCAAACATAA (mutations introduced are underlined). Mutations were confirmed by DNA sequencing. One microgram of the mutant plasmid was introduced into Mec-1 cells using the X-tremeGENE-HP DNA transfection reagent (Roche). Forty eight hours later, the cells were harvested and used for further experiments.

### Nuclear-cytosolic fractionation, immunoprecipitation and immunoblot analyses

CLL cell pellets were subjected to nuclear-cytosolic fractionation using NE-PER nuclear and cytoplasmic extraction kit (Pierce biotechnology, Rockford, IL). Immunoprecipitation of HDAC6, CDC37, p300 and HSP90 was performed as described previously [43, 54]. Immunoblot analyses were performed utilizing anti-HSF1, HSP90 alpha, HSP70, HSP27, BTK, c-RAF, CDK4, CDC37 and acetyl-lysine antibodies as previously described [43]. β-actin expression was used as a loading control. The data presented are representative of three independent experiments.

### Confocal immunofluorescent microscopy

CD19+ B cells were cytospun onto glass slides, fixed with 4% paraformaldehyde, treated with 0.5% Triton-X-100 and blocked with 3% BSA. The slides were incubated with HSF1 specific rabbit antibodies (Enzo Life Sciences, Inc., Farmigdale, NY), washed with PBS and HSF1 staining was visualized using secondary antibodies conjugated with Alexa Fluor-555. The slides were counterstained with DAPI to visualize the nucleus. Images are acquired using Pascal confocal microscope (Carl Zeiss) and processed with LSM-510 browser (Carl Zeiss) and Adobe Photoshop CS, as described previously [55].

### Cell death and cell cycle analysis

Cultured and primary CD19+ B cells were exposed to triptolide for 48 hours and stained with Annexin V-FITC and propidium iodide (PI). Apoptotic cells were determined by flow cytometric analysis by measuring the percentage of Annexin V and PI positive cells, as described previously [43]. The fraction of cells in G0-G1, S and G2-M phase of cell cycle were stained with PI and quantified as described previously, by flow cytometry [43].

### Molecular docking studies

Docking of triptolide to the HSP90-CDC37 complex was performed using AutoDock Vina software as described previously [56]. All predictions were made using the crystal structure of HSP90-CDC37 from the available protein data bank (PDB:2K5B) and the chemical structure of triptolide [39]. The best conformation was chosen with the lowest binding energy to make predictions about the hydrogen bonding between triptolide and the HSP90-CDC37 complex.

### *In vivo* Mec-1-luciferase cell xenografts

*In vivo* CLL xenografts were generated by injecting five million Mec-1-luciferase cells were injected into the tail vein of Rag2−/−γ(c)−/− immune deficient mice [47]. Mice were randomized and divided into 2 groups (*n* = 8) such that the average radiance was 1.00E+06 per group before they were treated minnelide. On day 7 after injection of cells, control mice received saline and the treatment group received daily intraperitoneal injection of minnelide at a dose of 0.42 mg/kg body weight for four weeks. Tumor burden was determined by *in vivo* bioluminescent imaging of the mice using a Xenogen IVIS 2000 *in vivo* imaging system (Caliper Life Sciences) every week. Mice were humanely sacrificed when they lost 15% of their body weight or when they showed signs of advanced disease, including hind limb paralysis. The day of death or euthanasia was noted and plotted on a Kaplan-Meier plot as previously described [55]. Molecular analysis of the effect of minnelide treatment was assessed by evaluating HSP90-dependent signaling pathways in the spleens of three mice from the minnelide-treated group (sacrificed on day 50) and compared with 3 control mice (spleens collected after euthanasia).

## SUPPLEMENTARY MATERIAL FIGURES


