# Supplementary material for: Targeting HSF1 disrupts HSP90 chaperone function in chronic lymphocytic leukemia
Source: Oncotarget. 2015 Sep 10;6(31):31767–79. doi: 10.18632/oncotarget.5167 (PMC4741638; doi:10.18632/oncotarget.5167)
Supplement: Supplementary file 1 [file oncotarget-06-31767-s001.pdf]

SUPPLEMENTARY FIGURES

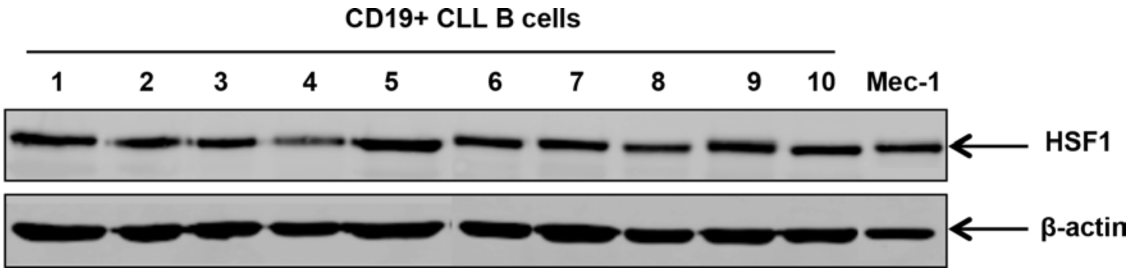

Supplementary Figure S1: CD19 + B cells lysates from CLL patients were immunoblotted for HSF1. β-actin was used as a loading control.

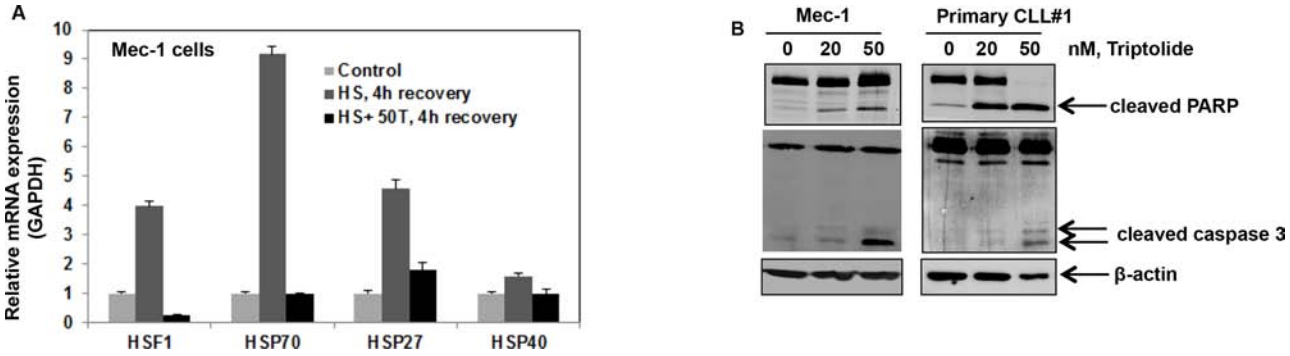

Supplementary Figure S2: A. Mec-1 cells were exposed to heat shock at 42°C for 1 hour (HS) and returned to 37°C for 4 hours, with our without the addition of triptolide. RNA was isolated from the resulting samples and they were reverse transcribed. q-PCR analysis was performed for the indicated mRNAs. GAPDH was used as an internal control. **B.** Mec-1 and Primary CLL cells were exposed to indicated-doses of triptolide and the expression of PARP and cleaved caspase 3 was assessed by immunoblot analysis. β-actin was used as loading control.

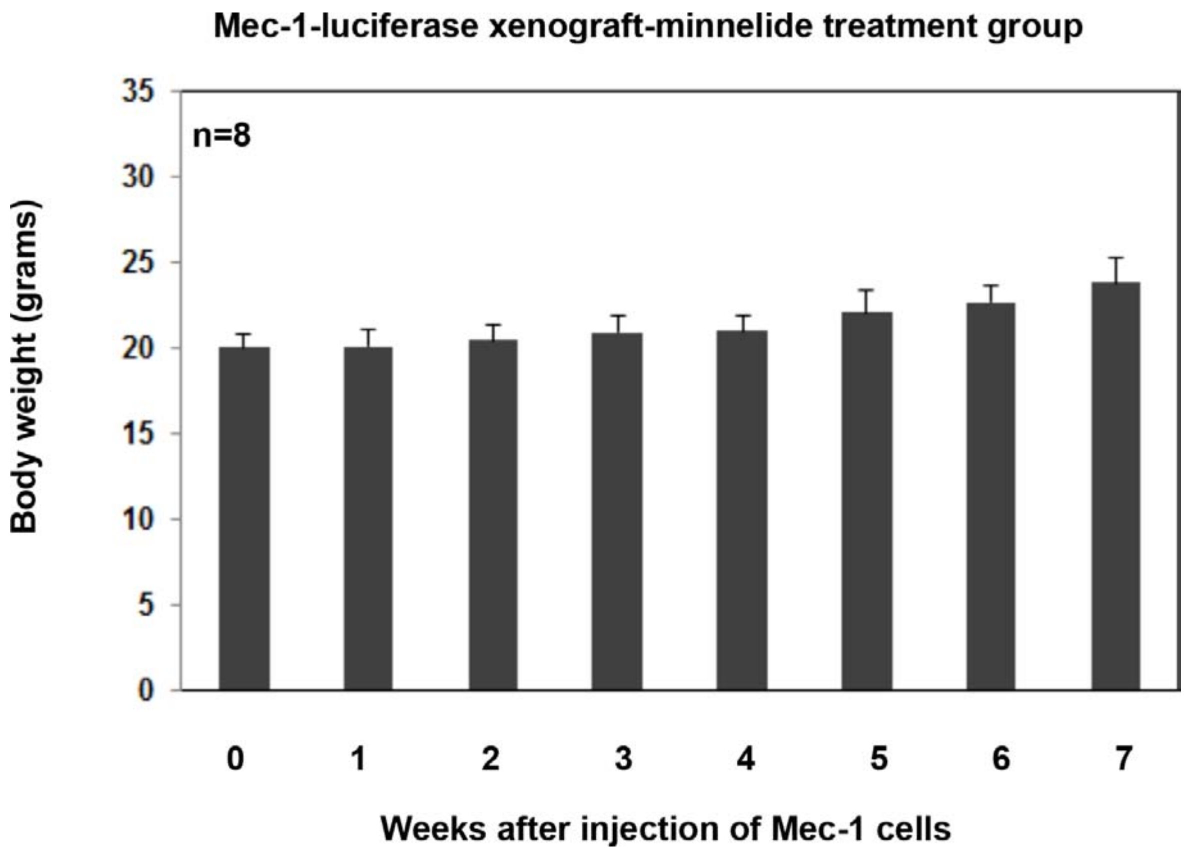

Supplementary Figure S3: Average body weights collected over a span of 7 weeks for the minnelide-treated mice.

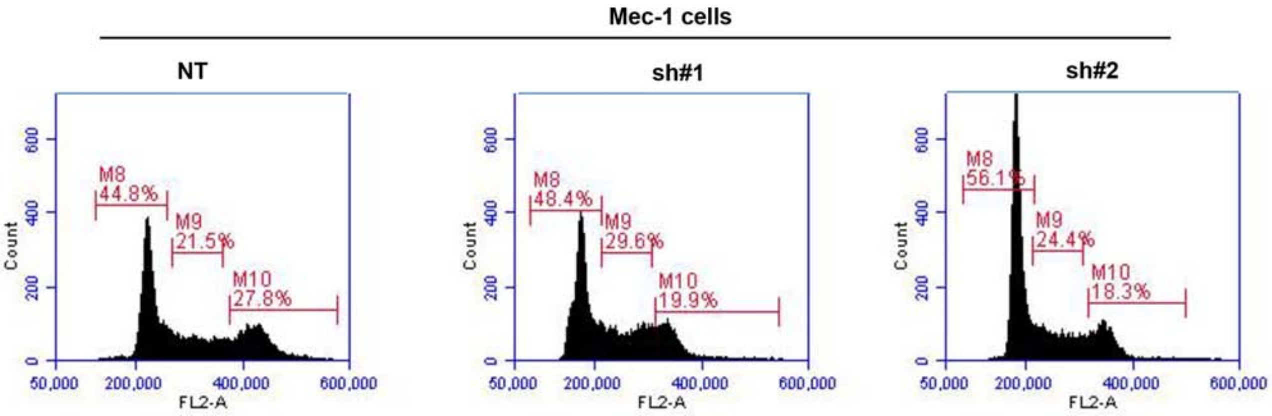

Supplementary Figure S4: Distribution of Mec-1 cells in G0-G1 (M8), G1-S (M9) and G2-M (M10) phase of cell cycle in non-targeted shRNA versus shHSF1-infected cells. FL2-A represents propidium iodide staining.
